# Consensus Guidelines on Human Epidermal Growth Factor Receptor 2 (HER2)-Low Testing in Breast Cancer in Malaysia

**DOI:** 10.3390/cancers16132325

**Published:** 2024-06-25

**Authors:** Pathmanathan Rajadurai, Sarala Ravindran, Bang Rom Lee, Suria Hayati Md Pauzi, Seow Fan Chiew, Kean Hooi Teoh, Navarasi S. Raja Gopal, Mastura Md Yusof, Cheng Har Yip

**Affiliations:** 1 Subang Jaya Medical Centre, Subang Jaya 47500, Malaysia; 2 Jeffrey Cheah School of Medicine and Health Sciences, Monash University Malaysia, Petaling Jaya 47500, Malaysia; 3 Department of Pathology, University of Malaya Medical Centre, Lembah Pantai, Kuala Lumpur 59100, Malaysia; 4 Premier Integrated Labs, Pantai Hospital Kuala Lumpur, Kuala Lumpur 59100, Malaysia; 5 Picaso Hospital, Petaling Jaya 46200, Malaysia; 6 Department of Pathology, Faculty of Medicine, Universiti Kebangsaan Malaysia Medical Centre, Kuala Lumpur 56000, Malaysia; 7 Sunway Medical Centre, Bandar Sunway, Subang Jaya 47500, Malaysia; 8 Putrajaya Hospital, Precinct 7, Putrajaya 62250, Malaysia; 9 Pantai Hospital Kuala Lumpur, Kuala Lumpur 59100, Malaysia

**Keywords:** breast cancer, HER2 testing, HER2-low scoring, consensus statement

## Abstract

**Simple Summary:**

At least 50% of breast cancers are estimated to belong to the ‘human epidermal growth factor receptor 2 (HER2)-low’ category. Since HER2-low breast cancer is amenable to targeted treatment with anti-HER2 antibody-drug conjugates, accurate assessment of patients’ HER2-low status is crucial for qualifying them for this treatment. Despite the 2018 American Society of Clinical Oncology and College of American Pathologists (ASCO/CAP) guidelines recognizing the clinical relevance of HER2-low testing, discrepancies in HER2 testing across laboratories globally hinder an accurate identification of HER2-low patients. To overcome these limitations, this consensus guideline aims to elucidate best practices for HER2 testing and HER2-low scoring in breast cancer. This guideline, while adapted to the Malaysian landscape, reflects recommendations from the latest international guidelines to guide pathologists in performing HER2 testing and address common issues faced in the Malaysian context, thus improving the quality of HER2 testing and ensuring the best available cancer care.

**Abstract:**

Breast cancer is one of the most common cancers in Malaysia. Recently, a new nomenclature was introduced for breast cancers with human epidermal growth factor receptor 2 (HER2) immunohistochemistry (IHC) 1+, or 2+ with negative in situ hybridization (ISH), i.e., HER2-low breast cancer. In current clinical practice, these breast cancers are reported as HER2-negative. Clinical trials have shown that HER2-low breast cancer benefits from targeted therapy with anti-HER2 antibody-drug conjugates. Unfortunately, various challenges and obstacles are faced by local pathologists in HER2 testing, which may jeopardize the standard of care for patients with HER2-low breast cancer. This consensus guideline aims to elucidate standard practices pertaining to HER2 testing and HER2-low interpretation in Malaysia. Topics discussed among a panel of local experts include tissue sampling and handling, assay and antibody selection, result interpretation and reporting, and quality assurance. Practice recommendations made in this consensus guideline reflect current international guidelines and, where appropriate, adapted to the Malaysian landscape.

## 1. Introduction

Breast cancer is the most diagnosed cancer worldwide, comprising a quarter of all cancer cases amongst women, and it was responsible for one in every six cancer deaths among women in 2020 [1]. It was the most common cancer in Malaysia between 2012 and 2016, accounting for nearly 20% of all cancer cases [2]. The overall five-year relative survival for breast cancer in Malaysia stands at 66.8% [3].

Breast cancer is subdivided into four clinical subtypes according to the presence/absence of hormone receptor (HR) and human epidermal growth factor receptor 2 (HER2): HR-positive/HER2-negative, HR-positive/HER2-positive, HR-negative/HER2-positive, and triple-negative breast cancer (TNBC). These subtypes hold prognostic relevance to survival and have been guiding treatment strategies for decades. HR-positive tumors express estrogen receptor (ER) and/or progesterone receptor (PR) and are eligible for endocrine therapy. The *HER2* oncogene encodes a membrane tyrosine kinase receptor that is overexpressed in about 15–20% of breast cancers, termed HER2-positive breast cancer. When overexpressed, potent proliferative and anti-apoptosis are activated, which become a major driver of tumor progression [4].

The 2018 American Society of Clinical Oncology and College of American Pathology (ASCO/CAP) HER2 testing guidelines recommend that HER2 status should be defined based on concurrent immunohistochemistry (IHC) and/or in situ hybridization (ISH) results [5]. A study of 2821 breast cancer patients in a tertiary hospital in Malaysia reported that 47.5% of patients were ER/PR-positive and HER2-negative, 15% were ER/PR/HER2-positive, and 17.9% were ER/PR-negative and HER2-positive, while 19.6% were ER/PR/HER2-negative [6]. Another study in Sarawak, Malaysia which analyzed data from 1034 patients, reported that 48% were ER/PR-positive and HER2-negative, 12% were ER/PR/HER2-positive, 11% were ER/PR-negative and HER2-positive, and 29% were ER/PR/HER2-negative. A higher incidence of ER/PR/HER2-negative breast cancer was seen among the native population of Sarawak [7]. In another study, the molecular profiling of 560 tumors from Malaysian patients revealed a higher prevalence of HER2-positive breast cancer in Malaysian women compared to Caucasian women [8].

Historically, HER2 overexpression was associated with higher rates of mortality compared to other molecular subtypes [9]. However, the development of anti-HER2 targeted therapy such as trastuzumab, pertuzumab, lapatinib and trastuzumab emtansine has significantly improved the prognosis of patients with HER2-positive breast cancer [4,10,11,12,13]. The management of most breast cancers traditionally defined as HER2-negative has now been transformed with the introduction of the new entity of ‘HER2-low’ breast cancer. These tumors express low levels of HER2, defined as tumors scored 1+ on IHC analysis, or tumors scored as 2+ on IHC and negative (non-amplified) on ISH [5,14,15]. At least 50% of breast cancers are anticipated to qualify as HER2-low [15].

### Current Treatment Options for HER2-Low Breast Cancer

The treatment landscape of HER2-low expressing tumors changed drastically with the introduction of a new generation of anti-HER2 antibody-drug conjugates (ADC). In 2022, the DESTINY-Breast04 trial reported trastuzumab deruxtecan (T-DXd) efficacy in patients with HER2-low metastatic breast cancer who were previously treated with one or two previous lines of chemotherapy [16]. Patients were randomized in a 2:1 ratio to receive T-DXd or physician’s choice chemotherapy (capecitabine, eribulin, gemcitabine, paclitaxel, or nab-paclitaxel). T-DXd resulted in improved progression-free survival and overall survival compared to chemotherapy regardless of HR status (positive or negative), and improved progression-free survival regardless of HER2-low status (IHC 1+ or IHC 2+/ISH-negative) [16]. The safety profile of T-DXd in the HER2-low subgroup was consistent with the established safety profile in patients with HER2-positive breast cancer [16]. The impressive outcomes of the DESTINY-Breast04 trial led to the approval of T-DXd by regulatory agencies as the first targeted therapy for HER2-low unresectable or metastatic breast cancer in patients who had received at least one prior line of chemotherapy in the metastatic setting or developed disease recurrence within 6 months of (neo)adjuvant chemotherapy [17].

Sacituzumab govitecan is another ADC that targets Trop-2, an epithelial antigen expressed by HR-positive/HER2-negative breast cancer and TNBC that is linked to tumor progression and poor prognosis [18]. It is approved for unresectable locally advanced or metastatic TNBC with two or more prior therapies, at least one of which in the metastatic setting, as well as for unresectable locally advanced or metastatic HR-positive/HER2-negative breast cancer, after failure of an endocrine-based therapy and at least two prior chemotherapies (based on the phase III ASCENT and TROPiCS-02 trial, respectively) [19,20,21]. The TROPiCS-02 trial demonstrated significant survival improvements with sacituzumab govitecan regardless of HER2 expression (IHC 0, IHC 1+ or IHC 2+/ISH−) [22].

The phase 2 DAISY trial evaluated T DXd in a similar patient population to DESTINY-Breast04, but also included HER2 IHC 0 tumors [23]. The trial showed that approximately 30.6% of patients with IHC 0 also demonstrated a response to T-DXd [24]. This is currently being evaluated by the ongoing DESTINY-Breast06 trial, which includes tumors expressing ultra-low levels of HER2 (IHC > 0 to <1+), to enhance our understanding of the definition of low HER2 expression and predictive benefit from HER2-targeted ADCs [25].

Various combination strategies and other therapies such as the monoclonal antibody margetuximab and three other anti-HER2 ADCs (trastuzumab duocarmazine, disitamab vedotin and MRG002) are also currently being investigated for their safety, tolerability, pharmacokinetics, and preliminary anti-tumor activity [26,27,28,29,30].

The increasing number of ADCs against HER2-low disease has made the accurate assessment of patients’ HER2-low status crucial to qualify these patients for targeted treatment [5]. In 2023, the ASCO/CAP published an update in their HER2 testing in breast cancer guidelines, recognizing the clinical relevancy of distinguishing IHC 0 from 1+ considering the new indication of T-DXd for HER2 IHC 1+ or IHC 2+/ISH-negative disease [31]. However, discordance in HER2 testing observed in laboratories globally in various reported clinical trials impedes an accurate identification of HER2-low patients [5,32]. Pre-analytical and analytical factors are an important cause of the discordance observed with HER2 testing. Furthermore, few guidelines specifically related to HER2-low testing are available. The local guideline on HER2 testing also warrants updating in line with developments in the ASCO/CAP testing guidelines [5,33]. To help overcome these limitations, this consensus guideline aims to elucidate the best practices pertaining to HER2 testing and HER2-low scoring in breast cancer.

## 2. Methods

This expert committee consists of seven pathologists, one oncologist and one breast surgeon, practicing in either private, government or university teaching hospital centers in Malaysia. The points of discussion and recommendation statements were made via discussion and review in three separate meetings held between April and June 2023. The recommendations agreed upon by the panel were collated, refined, and supported with the available literature. Multiple versions of the manuscript draft were shared with the expert committee for feedback and revisions. The final draft was unanimously agreed upon by all committee members.

## 3. Results

This consensus guideline is organized into seven Discussion sections (Discussions 1–7). Consensus recommendations made for each discussion topic are summarized in Table 1.

### 3.1. Discussion 1: Establishing HER2 Testing for Breast Cancer in Malaysia

HER2 testing in breast cancer has been documented in detail in the ASCO/CAP guidelines [5]. All patients must be tested for ER/PR and HER2 at the time of initial diagnosis of primary breast cancer, and HER2 must be tested regardless of ER/PR status [5]. The preferred type of biopsy for HER2 testing is a core needle biopsy if there is sufficient tumor tissue for assessment. Other tissue samples suitable for testing HER2 include wide local excision (WLE) or mastectomy specimens [34]. Fine needle aspiration cytology samples are less suitable for IHC testing [35].

When available, guided biopsy should be utilized, avoiding necrotic areas [36,37]. Core biopsy should also include normal breast tissue, which serves as an internal control when the tumor is ER/PR negative, and to ensure that normal breast tissue is HER2-negative [5]. If an internal control is not available within the biopsy sample, comments should be added to the report for testing to be repeated on the WLE or mastectomy specimen.

In patients who have received neoadjuvant therapy, HER2 testing should be repeated in cases where there is an incomplete pathological response. Patients with recurrent or metastatic disease should undergo a biopsy of the recurrent/metastatic tumor and repeat testing of ER, PR and HER2 status [5].

### 3.2. Discussion 2: Tissue Handling and Sample Preparation

Pre-analytical factors are an important source of test result discordance [38] and are especially difficult to control in outsourced material; this highlights the importance of adequate education of clinicians and adherence to a standardized guide in optimizing specimen processing for testing. Laboratory directors or persons in charge should ensure that pre-analytical issues are addressed, and corrective measures taken. Reducing errors in testing is critical as it impacts diagnosis and treatment. The recommended pre-analytical conditions for HER2 IHC are outlined in Table 2.

Tissue should be placed in an adequate volume of fixative (ideally 10:1 ratio of fixative to tissue) [42]. Delayed or prolonged formalin fixation may affect the antigenicity of the cancer cells; delaying fixation for more than 6 h or prolonging fixation for more than 10 days has been shown to affect HER2 IHC results by lowering the HER2 score [43]. Centers using rapid fixation and processing must validate their methodology for HER2 assessment [42]. Sections should be stained within 1–2 days of tissue sectioning and drying; long durations of section drying have been shown to cause a loss of HER2 expression. Freshly cut sections are recommended to either be dried at 60 °C for 1 h, or 37 °C overnight [42]. Tissue sections should ideally be used within 1 week of slide preparation, as significant variations in ambient temperature and humidity have been shown to affect immunoreactivity [44].

### 3.3. Discussion 3: Assay and Antibody Selection for HER2 Testing for Breast Cancer

Breast cancer HER2 status is assessed using IHC to determine HER2 protein expression, as well as by ISH to assess for *HER2* gene amplification. IHC should be performed as primary testing to evaluate HER2 protein expression status, as it is widely available in most pathology laboratories, is relatively inexpensive, and is the only recommended test available at the present time to detect HER2-low protein expression (tumor with IHC 1+ or IHC 2+ with non-amplified gene status). Chromogenic dyes are used in IHC tests to semi-quantitatively measure the overexpression of HER2 receptor on the tumor cells [45], while ISH encompasses several approaches, such as fluorescence in situ hybridization (FISH), chromogenic in situ hybridization (CISH) and silver-enhanced in situ hybridization (SISH) [4,46]. A dual-probe FISH assay is the preferred method of reflex *HER2* gene testing [5]. Both IHC and ISH testing are recommended to be performed on formalin-fixed, paraffin-embedded (FFPE) tissue section [4].

The inclusion of quality control tissue is highly recommended, ideally on each test slide or at least each batch of tests. The recommended control tissue is breast cancer with different IHC scores of 0, 1+, 2+ and 3+ and normal breast tissue as negative control [5]. The control material should preferably be similarly fixed and processed as the test tissue [42]. The use of cell line controls with full range of HER2 IHC expression enables optimal standardization and integrity of IHC test results, although the use of cell line controls is expensive.

#### 3.3.1. Detection of HER2 Protein Expression

The choice of assay and primary antibody is influenced by the availability of the recommended testing platforms and reagents in each institute. Commercial in vitro diagnostic devices (IVDs) should be utilized to standardize testing, maintain quality and to avoid variations [47,48]. Laboratory-developed tests (LDTs) are not recommended. If LDTs are used, the test results should be benchmarked and the laboratories performing the tests should participate in external quality assurance (EQA) schemes to ensure the integrity of testing and performance consistency.

HER2 IHC test should be performed using an FDA-approved assay or other validated assays [5]. Furthermore, the tests should also be performed by laboratories which have demonstrated proficiency in running these tests [5]. Two of the commonly utilized FDA-approved assays are the HercepTest (Dako, Glostrup, Denmark) and the HER2/neu (4B5) rabbit monoclonal primary antibody (Ventana, AZ, USA). Other FDA-approved HER2 antibodies include the CB11 Oracle (Leica Microsystems, Wetzlar, Germany) and the InSite Her-2/neu (CB11) (BioGenex Laboratories, Fremont, CA, USA) (Table 3).

Use of different antibodies for HER2 testing is a concern as the antibodies vary in performance characteristics, particularly the different sensitivities and specificities in the detection of low-HER2 expression status [53]. In October 2022, the Ventana 4B5 assay was approved by the US FDA as the first companion diagnostic test to identify HER2-low patients who may be eligible for targeted treatment [54]. It is notable that the recent DESTINY-Breast04 trial treated HER2-low patients based on IHC results obtained using the Ventana 4B5 assay [16]. The recent ESMO expert consensus stated that the Ventana 4B5 assay identified a higher proportion of HER2-low cases compared to HercepTest SK001, whereas HercepTest GE001 picked up more HER2-low cases than Ventana 4B5 [55,56,57]. In addition, a comparison study between HercepTest GE001 and Ventana 4B5 using 119 preselected breast cancer samples demonstrated that both IHC assays are highly suitable for the detection of HER2 protein, while fewer assay-related failures were observed using HercepTest [57].

As the HER2 IHC score is a semi-quantitative assessment, it may not be adequately sensitive to detect low levels of HER2 [15]. Newer tests designed to specifically address and improve the identification of HER2-low cases are desirable. Among such tests would be attempting to quantify mRNA copy number via reverse transcription polymerase chain reaction (RT-PCR) and next-generation sequencing (NGS). Other testing methods such as immunofluorescence-based automated quantitative analysis methods and quantitative IHC methods are under development. All these tests need extensive analysis and appropriate validation prior to being approved for diagnostic use [58].

#### 3.3.2. Detection of HER2 Gene Amplification

For tumors tested as HER2 2+ on IHC, reflex ISH testing should be performed [5]. In case of doubt, HER2 IHC scores other than 2+ may also undergo ISH for confirmation of *HER2* gene status. This may be advantageous as the cost of ISH is small compared to the overall costs of treatment (financial costs, as well as ‘cost’ of adverse effects from treatment). However, ISH should not be performed on its own as it will lead to the underdiagnosis of HER2-low cases, specifically HER2 IHC 1+ cases. Nonetheless, ISH may be uninterpretable in a biopsy sample with very limited numbers of invasive cancer cells, usage of fixative other than buffered formalin, over-fixation for more than 48 h or auto-fluorescent background [4]. The different types of ISH tests, their principles, advantages, and disadvantages are summarized in Table 4.

Examples of FDA-approved ISH test kits are listed in Table 5.

There are several additional tests that can potentially be utilized for HER2 testing, such as the protein proximity assay and reverse transcription quantitative polymerase chain reaction (RT-qPCR). However, it is imperative to note that these tests are yet to be approved for routine diagnostic testing in current guidelines. The protein proximity assay, HERmark™ Breast Cancer Assay (Monogram Biosciences, San Francisco, CA, USA), can be used on FFPE tissue samples. It quantifies the total amount of cellular protein or two similar or dissimilar proteins in proximity. This test has been utilized to quantify HER2 protein level and the amount of HER2 homodimers in breast cancer tissue [4]. RT-qPCR, on the other hand, can be used to detect *HER2* gene amplification in FFPE breast cancer tissue. It can be utilized in combination or as an alternative to the widely used IHC and ISH methods, particularly in cases in which HER2 status was uncertain with IHC and ISH [15,64]. This method is advantageous in that it gives a standardized, objective, and automated evaluation [15], as the polymerase chain reaction (PCR) result may not be as readily influenced by evaluator errors such as those seen with IHC and ISH [65].

### 3.4. Discussion 4: Results’ Interpretation

#### 3.4.1. HER2 IHC Scoring

The HER2 IHC scoring according to the ASCO/CAP HER2 Testing in Breast Cancer 2018 Update is shown in Table 6. The interpretation of HER2 IHC results should be based on established guidelines, as results near cut-off values (i.e., at the 10% mark) and intuitive interpretation of staining intensities may lead to variability in the scoring for IHC 0/1+/2+. There have also been updates to the ASCO/CAP guidelines, with cut-offs of tumor cells ranging from 30% (in differentiating between 2+ and 3+) in the 2007 guideline to 10% in the 2013 and 2018 guidelines [5,34,38]. In addition, the 2007 and 2013 ASCO/CAP guidelines included an ISH equivocal category, which posed problems when making clinical decisions [34,38,66].

#### 3.4.2. HER2 ISH Testing

The 2018 ASCO/CAP guidelines provide detailed descriptions on the evaluation of *HER2* gene amplification by ISH assay (both single- and dual-probe ISH) [5]. The guidelines recommend that laboratories using single-probe ISH assays include a concomitant IHC review as part of the interpretation of all ISH assay results; hence, the HER2 status can be designated to positive or negative without an equivocal category [5]. For dual-probe ISH, implementation of the updated 2018 ASCO/CAP guidelines has led to significant increase of HER2-negative rates through the reclassification of ISH equivocal cases from the 2007/2013 guidelines [5,34,38]. HER2 status designation by using a concomitant IHC review in ISH group 2 (*HER2*/chromosome enumeration probe 17 [CEP17] ratio ≥2.0; average *HER2* copy number <4.0 signals/cell), ISH group 3 (*HER2*/CEP17 ratio <2.0; average *HER2* copy number ≥6.0 signals/cell), and ISH group 4 (*HER2*/CEP17 ratio <2.0; average *HER2* copy number ≥4.0 and <6.0 signals/cell) is clearer and eliminates the ISH equivocal category [66].

#### 3.4.3. Potential Challenges in Results’ Interpretation

HER2 intratumoral heterogeneity is a well-recognized phenomenon in breast cancer. Although its frequency varies, it has been reported to occur in up to 40% of breast cancers [67,68]. In 2009, a supplement to the 2007 ASCO/CAP guidelines defined HER2 heterogeneity as the presence of 5% to 50% of total tumor cells with *HER2* gene amplification (*HER2*/CEP17 ratios of more than 2.2) [69]. Following this, the 2013 and 2018 ASCO/CAP guidelines defined intratumoral heterogeneity as the presence of a second population of tumor cells, of which 10% or more were tumor cells have a different *HER2* copy number and/or *HER2*/CEP17 ratio; the guidelines recommended additional counting and reporting of at least 20 cells in the second tumor population [5,34].

As intratumor heterogeneity may be a cause of variability, an IHC result of 0 on a biopsy specimen may warrant re-testing on the excision specimen [5]. An evaluation should be carried out by assessing the average *HER2* copy number and *HER2*/CEP17 ratio separately for amplified and non-amplified areas in each tumor population [66]. The percentage of the tumor population with amplification should also be provided to minimize interobserver variability [65]. When assessing the tumor by FISH, an evaluation of at least two (and up to four) representative fields of the invasive tumor should be performed [69]. In case of equivocal HER2 IHC results, reflex ISH may be ordered for confirmation. However, reflex ISH for IHC 2+ results will incur additional cost, consume resources, and increase the turnaround time [70].

The HER2 status can change following neoadjuvant therapy, cancer recurrence or differ between metastatic sites. Therefore, IHC and ISH slides should be evaluated concurrently to determine the presence and distribution of heterogeneity for HER2. In addition, pathologists should be aware that CEP17 copy number gain may lead to an underestimation of HER2 status [66].

Another diagnostic challenge in the interpretation of HER2 IHC lies at the 10% cut-offs between the different scores, especially between 1+ and 2+. Unless every equivocal IHC result is tested with ISH, it may be challenging to achieve perfect concordance between IHC and ISH. The limitation of the IHC test in determining scores of 1+ and 2+ (in certain instances due to ambiguity between 1+ or 2+, suspected poor specimen integrity, presence of cytoplasmic staining or insufficient intensity and completeness of membrane staining) may lead to discrepancies in the reporting, and an increase in the reporting of tumors as HER2 2+, thus leading to an increase in reflex ISH testing. This highlights the importance of participating in EQAs and consultation with peers to ensure consistency of HER2 reporting and prevent drift in testing results (Discussion 7). Apart from interpretation variabilities, discordance may also arise from specimen handling, pre-analytical conditions and the type of antibodies used [5].

#### 3.4.4. Recommended Practice in Results’ Interpretation

Laboratories in Malaysia are recommended to score HER2 IHC based on the updated 2018 ASCO/CAP guideline. Occasionally, pathologists may encounter unusual staining patterns of HER2 by IHC, which are not discussed within the ASCO/CAP guidelines [5]. Examples include moderate-to-intense but incomplete (basolateral or lateral) staining, or circumferential membrane IHC staining that is intense but in ≤10% of tumor cells [5]. In such cases, it may be prudent to consider the IHC result as 2+ (equivocal) and reflex to ISH testing to determine HER2 status.

Algorithms for evaluating HER2 protein expression by IHC and *HER2* gene amplification by ISH are outlined in the ASCO/CAP guideline [5]. IHC scoring using the magnification rule is recommended to circumvent the restrictions of human vision. As a guide, IHC 1+ cannot be reliably assessed using a 10× objective and is best observed using a 40× objective. IHC 2+ is usually visible using a 10× objective and cannot be reliably assessed using a 5× objective. On the other hand, IHC 3+ staining is usually visible using a 5× objective [71]. According to the ASCO/CAP guideline, an IHC 3+ result should be visible at low magnification (2× or 4× objectives) and observed within a homogeneous and contiguous invasive cell population [5].

Strict adherence to recommended guidelines is necessary to prevent variability in interpretation; however, unusual staining patterns and staining patterns close to cut-off values will likely be seen in real-life practice. Interpretation in these cases would still depend on a pathologist’s judgement and a certain degree of subjectivity is inevitable, which may be resolved by re-testing, interdepartmental consultation, or orthogonal testing.

In future, digital image analysis of HER2 IHC may become a valid tool to determine HER2 scores for laboratories with access to such platforms. Optimal calibration and validation by individual laboratories are recommended as different platforms use different algorithms and approaches to classifying tissues and cellular components, thereby leading to the possibility of inter-platform variability in scoring [72]. The College of American Pathologists (CAP) has released updated guidelines on validating whole-slide imaging systems for diagnostic purposes [73]. There are currently no specific published guidelines available on the validation of image-based artificial intelligence algorithms, although a short discussion on this topic has been made (https://www.cap.org/member-resources/clinical-informatics-resources/how-to-validate-ai-algorithms-in-anatomic-pathology (accessed on 12 May 2024)).

**Figure 1:**
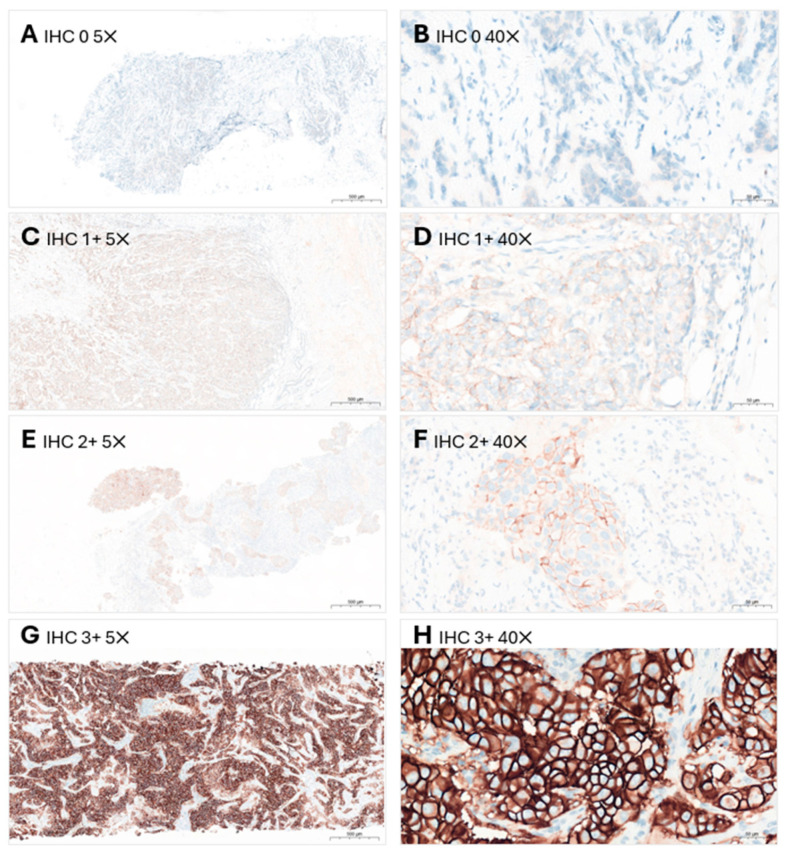
HER2 immunoscoring in breast cancer using the Ventana 4B5 assay. HER2 IHC 0 is shown at (**A**) 5× objective and (**B**) 40× objective (absent to barely perceptible staining in ≤10% of the invasive malignant cells); HER2 IHC 1+ is shown at (**C**) 5× objective and (**D**) 40× objective (faint-to-weak, incomplete membrane staining in >10% of invasive malignant cells); HER2 IHC 2+ is shown at (**E**) 5× objective and (**F**) 40× objective (weak-to-moderate, complete membrane staining in >10% of invasive malignant cells); HER2 IHC 3+ is shown at (**G**) 5× objective and (**H**) 40× objective (circumferential membrane staining that is complete, intense and in >10% of invasive malignant cells, visible at low magnification levels of 4×/5×). HER2: human epidermal growth factor receptor 2; IHC: immunohistochemistry.

**Figure 2:**
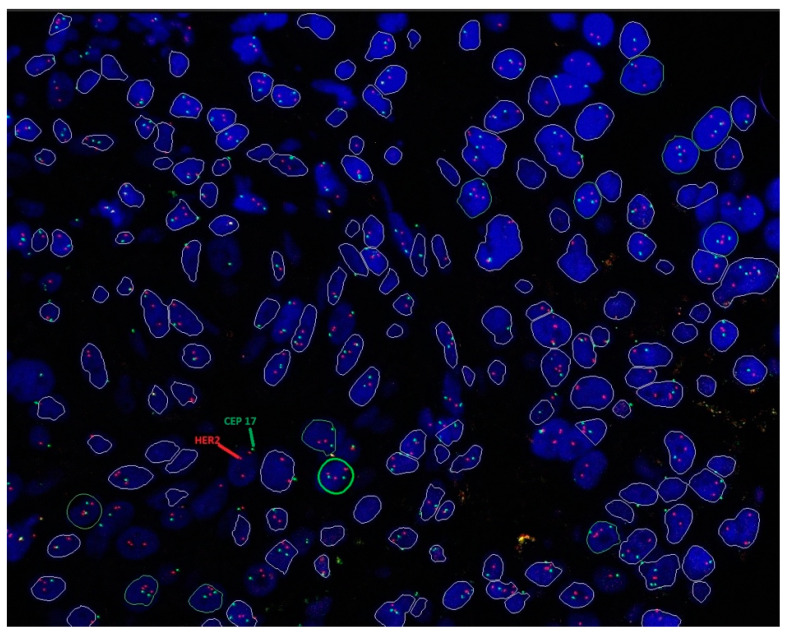
*HER2* gene status in breast cancer identified using the dual-probe FISH assay. The sample showed a tumor cell [green circle] with a *HER2*/CEP17 ratio of 1.3 and an average HER2 copy number of 2.7, placing the tumor under ISH group 5 (HER2-negative). CEP17: chromosome enumeration probe 17; ISH: in situ hybridization; FISH: fluorescence in situ hybridization; HER2: human epidermal growth factor receptor 2.

Sample images of HER2 IHC and ISH testing are shown in Figure 1 and Figure 2, respectively.

### 3.5. Discussion 5: Results Reporting

A HER2 status report is essential to guide patient management and prognosis. HER2 evaluation could be determined by using IHC or ISH methods. When both IHC and ISH are performed on the same tumor, the results should be correlated.

#### 3.5.1. Reporting HER2 IHC Score

Guideline-recommended components for reporting HER2 IHC score are shown in Table 7.

#### 3.5.2. Reporting HER2 ISH Result

Determination of *HER2* gene amplification according to the dual-probe assay method requires a record of the average number of *HER2* signals/cell, the average number of CEP17 signals/cell, and the *HER2*/CEP17 ratio [74]. HER2 ISH status (positive or negative) is determined according to Table 8. On the other hand, the single-probe assay method requires only the average number of *HER2* signals/cell [5].

#### 3.5.3. Reporting Heterogenous Tumors

In case of intratumor heterogeneity, the average *HER2* copy number and *HER2*/CEP17 ratios in the amplified and non-amplified areas must be evaluated separately for each tumor sub-population. The percentage of the total tumor population with amplification should also be reported [66].

### 3.6. Discussion 6: Overcoming Obstacles in HER2 Testing

The most important barriers to HER2 testing are inadequate funding, high cost of testing, and inequitable distribution of resources and services. While HER2 by IHC is available in most laboratories, reflex testing by ISH is not readily available due to additional cost and infrastructure requirements. Most laboratories resort to outsourcing ISH testing to central laboratories, which delays the turnaround time. Unfortunately, samples outsourced to central laboratories for IHC and/or ISH testing run the risk of being over-fixed due to prolonged storage and time spent in transit. Increasing the number of central testing facilities is required to improve the turnaround time. Furthermore, IHC/ISH testing should also ideally be made available in large hospitals with a histopathology diagnostic service, with adequate manpower and infrastructure to ensure testing accuracy. This would help establish a turnaround time of 7–10 working days. However, the costs of infrastructure (testing facilities, machines) and manpower (pathologists, technicians) needed to set up such a service remain a significant barrier.

Additionally, pre-analytical factors such as fixation, antigen retrieval, antibody clones, enzymatic activity, reaction time, temperature and substrate concentration may also form obstacles in HER2 testing and influence HER2 staining intensity. Laboratory personnel should play an active role in maintaining quality and ensure that pre-analytical issues are appropriately addressed.

Advances in the diagnosis and treatment of patients with breast cancer require clinicians to stay up to date to ensure optimal diagnosis and clinical management of patients. In the area of HER2 testing, workshops for pathologists are essential as the correct interpretation of HER2 status is vital to ensure that breast cancer patients receive optimal treatment, which in turn would improve outcomes. At the same time, workshops to educate clinicians and related medical personnel on the importance of pre-analytical factors and how these impact the result interpretation are an important step in minimizing such errors.

Many Malaysian patients lack access to life-saving anti-HER2 therapy. In 2014, only 19% of patients with HER2-positive tumors received trastuzumab in both the public and private healthcare system despite it being considered an essential medicine by the World Health Organization (WHO) with an established clinical benefit in early breast cancer [75]. Enhancing the delivery of cancer services in the country is a constant challenge as many of the issues outlined above must be continuously addressed.

### 3.7. Discussion 7: Upholding Quality in HER2 Testing

Internal and external audits, appropriate documentation of testing procedures and protocols, peer review, and monitoring of HER2 positivity rates are all important quality measures in HER2 testing.

The impact of pre-analytical factors on tissue quality and the final testing result must also be emphasized to all members involved in the care of breast cancer patients. Internal audits allow for continuous monitoring of the reporting of HER2 positivity in a department. In addition, workshops and assessments (International Organisation for Standardisation [ISO] certification) for laboratory personnel help maintain competency in the laboratory [5]. A study assessing the discrepancies in HER2 testing between unaccredited local laboratories and an accredited central testing center reported that 85% of IHC 0 scored in local laboratories were IHC 1+ or 2+, suggesting the role of laboratory accreditation in distinguishing subgroups of HER2-low breast cancer [76].

Moreover, participation in EQA programs such as the Royal College of Pathologists of Australasia Quality Assurance Programme (RCPAQAP) or the Nordic Immunohistochemical Quality Control (NordiQC) ensures accuracy and consistency in testing and reporting. Participation may start with international EQA programs before transitioning to local EQA programs as the body of locally available knowledge grows.

Furthermore, the establishment of an institutional database is an important quality assurance measure to monitor the frequency of HER2 positivity, and to track possible deviations in HER2 positivity scores. In addition to an institutional or laboratory-based database, a national database for HER2 positivity is also desired.

## 4. Conclusions

Accurate diagnosis of patients’ HER2-low status is crucial to qualify these patients for targeted treatment. However, discordance in HER2 testing impedes accurate diagnosis of HER2-low patients. This consensus guideline has been established as a guide for pathologists in performing HER2 testing, and addresses common issues faced with HER2 testing in the local Malaysian context. The present guidelines provide substantial updates to the previous Malaysian guidelines [33] by incorporating new evidence and technological advancements to provide clear, precise, and comprehensive recommendations. These guidelines emphasize the importance of accurate diagnostic procedures, the role of novel therapies, and the need for continuous quality improvement in testing practices. The changes aim to enhance the diagnostic accuracy and therapeutic outcomes for patients with HER2-positive and HER2-low breast cancer.

We hope that this consensus guideline will provide the necessary guidance to improve the quality of HER2 testing performed in Malaysia and help Malaysian patients receive the best available cancer care. These Malaysian guidelines are current and aligned with the most recent ASCO/CAP guidelines [31]. To the best of our knowledge, there are no similar guidance documents locally available on HER2 testing in breast cancer. Therefore, the authors are hopeful that these local guidelines might also prove useful regionally, providing guidance to healthcare practitioners involved in the management of breast cancer patients.

## Figures and Tables

**Table 1:** Summary of consensus recommendations for HER2 testing in breast cancer.

Discussion 1: Establishing HER2 testing for breast cancer in Malaysia
HER2 testing should be performed at the time of initial diagnosis of primary breast cancer in all patients. Core needle biopsy is the preferred specimen for HER2 testing. Guided biopsy should be utilized whenever possible. Where possible, the core biopsy should include normal breast tissue to serve as an internal control. In post-neoadjuvant cases, HER2 testing should be repeated when there is an incomplete pathological response. Patients with recurrent or metastatic disease should undergo repeat testing on the recurrent/metastatic tumor.
**Discussion 2: Tissue handling and sample preparation**
Adequate clinician education and adherence to standard guidelines is essential to optimize specimen processing for testing. Pre-analytical issues should be addressed and rectified whenever possible, to reduce errors and lead to better decisions in diagnosis and treatment.
**Discussion 3: Assay and antibody selection for HER2 testing for breast cancer**
IHC and ISH testing should be performed on FFPE tissue sections using an FDA-approved or other validated commercial IVDs, to maintain quality and to minimize errors in interpretation. The use of LDT is not recommended. The use of control tissue with IHC scores of 0, 1+, 2+ and 3+ and normal breast tissue as negative control is mandatory. A dual-probe FISH assay is the preferred method of reflex *HER2* gene testing. Other properly validated ISH methods, i.e., CISH, SISH and DDISH may also be utilized. Utilizing ISH testing alone as the only test to determine HER2 status is not recommended as HER2-low cases (IHC 1+, and IHC 2+/ISH-negative cases) will be missed.
**Discussion 4: Result interpretation**
Interpretation of HER2 IHC and ISH test results should be based on current established guidelines. In cases where ambiguous HER2 IHC results are observed, reflex ISH may be ordered for confirmation.
**Discussion 5: Results reporting**
Reporting of HER2 IHC and ISH test results should be based on current established guidelines. Reports should be clear and concise, and preferably standardized between laboratories.
**Discussion 6: Overcoming obstacles in HER2 testing**
HER2 IHC testing should ideally be made available in large hospitals with histopathology diagnostic services, with adequate manpower and infrastructure to ensure testing accuracy. This would help establish a turnaround time of 7–10 working days. Clinician education is necessary to emphasize the importance of proper sample collection and fixation-related protocols, to minimize or eliminate the impact of adverse pre-analytical factors.
**Discussion 7: Upholding quality in HER2 testing**
The following strategies may be taken to ensure quality in HER2 testing: Regular surgeon, radiologist, and oncologist education on the impact of pre-analytical factors during sample collection, fixation, and transport to the laboratory. Regular laboratory personnel assessment and workshops. Maintaining appropriate documentation of testing procedures and protocols. Obtaining laboratory accreditation such as ISO certification. Participation in EQA programs. Establishment of institutional and national database(s) to monitor the frequency of HER2 positivity, and to track possible deviations in HER2 positivity scores.

CISH: chromogenic in situ hybridization; DDISH: dual-color dual-hapten in situ hybridization; EQA: external quality assurance; FDA: Food and Drug Administration; FFPE: formalin-fixed, paraffin-embedded; FISH: fluorescence in situ hybridization; HER2: human epidermal growth factor receptor 2; IHC: immunohistochemistry; ISH: in situ hybridization; ISO: International Organisation for Standardisation; IVD: in vitro diagnostic devices; LDT: laboratory-developed test; SISH: silver-enhanced in situ hybridization.

**Table 2:** Recommended pre-analytical conditions for HER2 IHC testing [4,5,39,40,41].

Parameter	Recommendation
Cold ischemia time	As short as possible (one hour or less)
Fixative	10% neutral buffered formalin
Time of fixation	6–72 h
Preparation	Paraffin-embedded; sliced at 5- to 10-µm intervals
Use of tissue section	Should ideally be used within 1 week of slide preparation
Storage time for tissue sections	Sections should be stored in a closed box at 2–8 °C, and should be used within 6 weeks
Storage condition and time for FFPE blocks	Should be protected from light, heat, and humidity. The period of storage should comply with local regulatory requirements (20 years in Malaysia)
Decalcification	When decalcification is performed, EDTA should be used, and strong acids should be avoided

EDTA: ethylenediaminetetraacetic acid; FFPE: formalin-fixed and paraffin-embedded; HER2: human epidermal growth factor receptor 2; IHC: immunohistochemistry.

**Table 3:** US FDA-approved antibodies utilized for HER2 IHC testing.

Antibody	Description	Platform
PATHWAY anti-HER2/neu (4B5) [49]	Rabbit monoclonal primary antibody	Ventana BenchMark ULTRA IHC/ISH System
HercepTest pharmDx [50]	Rabbit monoclonal primary antibody	Dako Omnis, Automated Link Platforms, Dako Autostainer or manually
CB11 Oracle [51]	Mouse monoclonal primary antibody	Leica Bond Oracle HER2 IHC System
InSite Her-2/neu (Clone CB11) [52]	Mouse monoclonal primary antibody	BioGenex i6000™ Automated Staining System and the Optimax^®^ Plus Consolidated Staining System

HER2: human epidermal growth factor receptor 2; IHC: immunohistochemistry; ISH: in situ hybridization; US FDA: United States Food and Drug Administration.

**Table 4:** The principles, advantages, and disadvantages of different types of ISH tests [4].

Type of ISH	Principle of Test	Advantages	Disadvantages
CISH	Indirect labeling using chromogens	Produces a permanent stain. Easy identification of target cells and tumor heterogeneity. Can be viewed using light microscopy. CISH slides can be archived.	Does not allow for definitive determination of *HER2* gene copy number.
FISH	Fluorescent-labeled DNA probes that produces colored signals	Sensitive method.	Can only be viewed using fluorescent microscopy. Fluorescent signals are not permanent and fade over time. Evaluation requires trained personnel, a high-resolution digital camera and time-consuming manual counting.
SISH	Indirect labeling using DNP-labeled probes	Produces a permanent stain. Can be viewed using light microscopy. Slides can be archived. Allows quantitative scoring of gene copy number.	-
DDISH	Utilizes both silver and chromogen detection systems.	Produces a permanent stain. Can be viewed using light microscopy. Slides can be archived. Easy comparison and correlation of morphological findings, as it can be read with H&E-stained slide in the same settings.	-

CISH: chromogenic in situ hybridization; DDISH: dual-color dual-hapten in situ hybridization; DNA: deoxyribonucleic acid; DNP: dinitrophenyl; FISH: fluorescence in situ hybridization; H&E: hematoxylin and eosin; HER2: human epidermal growth factor receptor 2; ISH: in situ hybridization; SISH: silver-enhanced in situ hybridization.

**Table 5:** FDA-approved ISH test kits for confirmation of *HER2* gene status.

Type of ISH	FDA-Approved Test Kit
CISH	SPOT-Light HER2 CISH kit (Invitrogen, Waltham, MA, USA) [59] HER2 CISH pharmDx™ Kit (Dako, Glostrup, Denmark) [60]
FISH	PathVysion (Vysis, Abbott Laboratories, Chicago, IL, USA) [61] HER2 IQFISH pharmDx (Dako, Glostrup, Denmark) [62]
DDISH	VENTANA HER2 Dual ISH DNA Probe Cocktail (Ventana, Oro Valley, AZ, USA) [63]

CISH: chromogenic in situ hybridization; DDISH: dual-color dual-hapten in situ hybridization; DNA: deoxyribonucleic acid; FISH: fluorescence in situ hybridization; FDA: Food and Drug Administration; HER2: human epidermal growth factor receptor 2; ISH: in situ hybridization.

**Table 6:** HER2 IHC scoring based on ASCO/CAP HER2 Testing in Breast Cancer 2018 Update [5].

IHC Score	ASCO/CAP Guidelines Definition
0	No staining observed or membrane staining that is incomplete and is faint or barely perceptible and in ≤10% of the invasive tumor cells.
1+	Incomplete membrane staining that is faint or barely perceptible and in >10% of the invasive tumor cells.
2+	Invasive breast cancer with weak to moderate complete membrane staining observed in >10% of tumor cells. Test result is reported as equivocal and must undergo reflex ISH test.
3+	Circumferential membrane staining that is complete, intense and in >10% of tumor cells.

ASCO/CAP: American Society of Clinical Oncology and College of American Pathology; HER2: human epidermal growth factor receptor 2; IHC: immunohistochemistry; ISH: in situ hybridization.

**Table 7:** Reporting HER2 IHC score [5,74].

HER2 IHC Result	IHC Score
Negative	Score 0 (no staining observed or membrane staining that is incomplete and is faint or barely perceptible and in ≤10% of the invasive tumor cells)
	Score 1+ * (incomplete membrane staining that is faint or barely perceptible and in >10% of the invasive tumor cells)
Equivocal	Score 2+ * (invasive breast cancer with weak to moderate complete membrane staining observed in >10% of tumor cells)
Positive	Score 3+ (circumferential membrane staining that is complete, intense and in >10% of tumor cells)

HER2: human epidermal growth factor receptor 2; IHC: immunohistochemistry. * Breast cancers with HER2 IHC scores 1+ or 2+ and a negative ISH result are eligible for clinically appropriate HER2-targeted therapy and may now be reported as HER2-low.

**Table 8:** Reporting HER2 ISH status, according to the ASCO/CAP HER2 Testing in Breast Cancer 2018 Update [5,66].

HER2 ISH Status	Criteria (Dual-Probe Assay)
Positive (amplified)	*HER2*/CEP17 ratio ≥2.0 and average *HER2* copy number ≥4.0 signals/cell (group 1)
	*HER2*/CEP17 ratio ≥2.0 and average *HER2* copy number <4.0 signals/cell (group 2) with concurrent IHC 3+
	*HER2*/CEP17 ratio <2.0 and average *HER2* copy number ≥6.0 signals/cell (group 3) with concurrent IHC 2+ ^a^
	*HER2*/CEP17 ratio <2.0 and average *HER2* copy number ≥6.0 signals/cell (group 3) with concurrent IHC 3+
	*HER2*/CEP17 ratio <2.0 with average *HER2* copy number ≥4.0 and <6.0 signals/cell (group 4) with concurrent IHC 3+
Negative (non-amplified)	*HER2*/CEP17 ratio <2.0 with average *HER2* copy number <4.0 signals/cell (group 5)
	*HER2*/CEP17 ratio ≥2.0 and average *HER2* copy number <4.0 signals/cell (group 2) with concurrent IHC 2+ ^b^
	*HER2*/CEP17 ratio <2.0 with average *HER2* copy number ≥4.0 and <6.0 signals/cell (group 4) with concurrent IHC 2+ ^b^
	Groups 2, 3, and 4 with concurrent IHC 0 or 1 +

ASCO/CAP: American Society of Clinical Oncology and College of American Pathology; CEP17: chromosome enumeration probe 17; IHC: immunohistochemistry; ISH: in situ hybridization; HER2: human epidermal growth factor receptor 2. ^a^ A second reviewer blinded to previous result re-counts ISH. If the repeated ISH result is categorized to the same group, it is regarded as HER2-positive. ^b^ A second reviewer blinded to previous result re-counts ISH. If the repeated ISH result is categorized to the same group, it is regarded as HER2-negative.

## Data Availability

Data sharing is not applicable.
